# Supplementary material for: Association between oral microbiome and seven types of cancers in East Asian population: a two-sample Mendelian randomization analysis
Source: Front Mol Biosci. 2023 Nov 21;10:1327893. doi: 10.3389/fmolb.2023.1327893 (PMC10702768; doi:10.3389/fmolb.2023.1327893)
Supplement: Supplementary file 1 [file DataSheet1.ZIP › Supplementary Materials/MR plots of saliva/saliva═╝/Lung Cancer/pheno.185_to_lung cancer_leave_one_out.pdf]

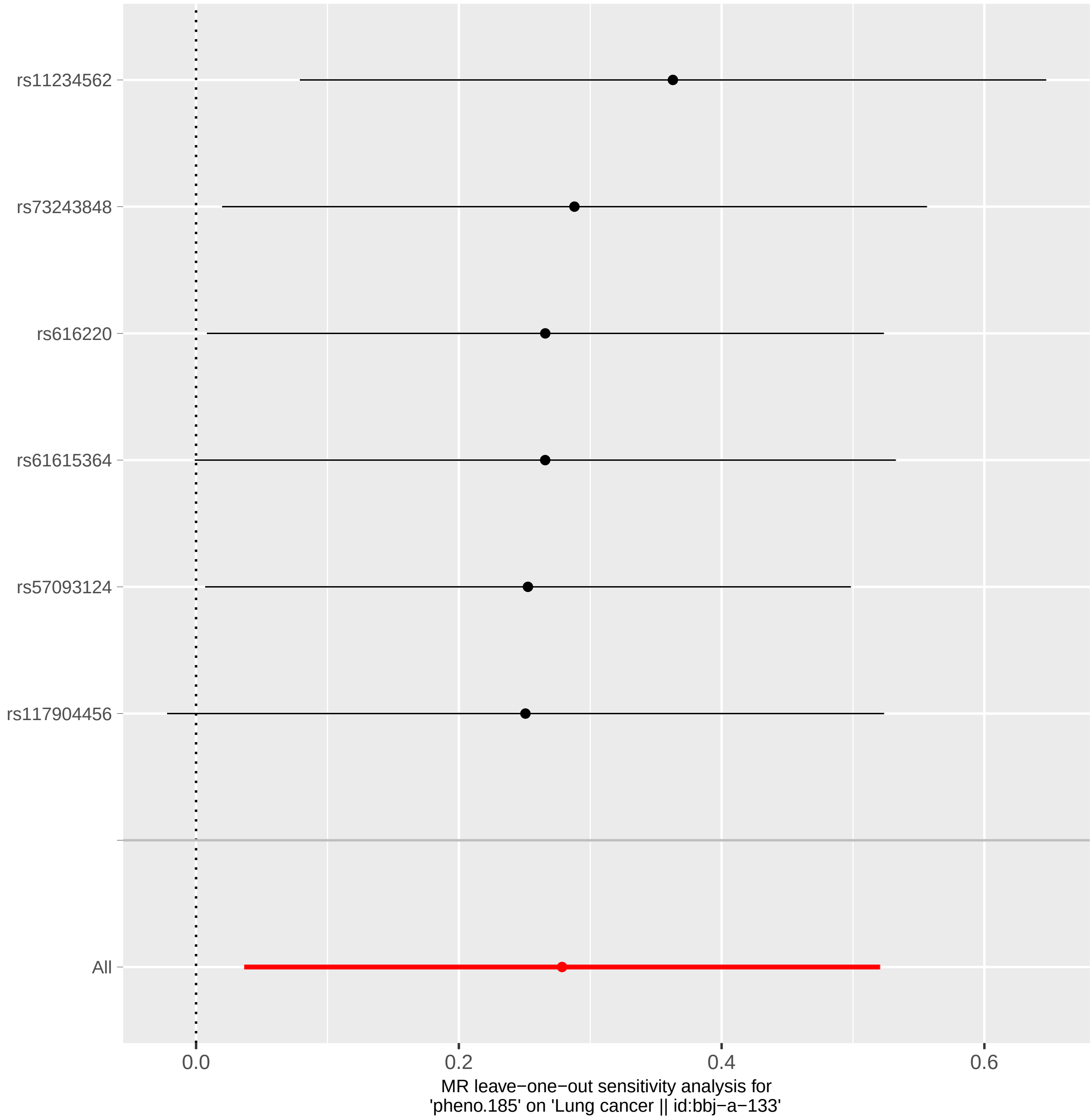
rs11234562
rs73243848
rs616220
rs61615364
rs57093124
rs117904456
All
0.0
0.2
0.4
0.6
MR leave−one−out sensitivity analysis for
'pheno.185' on 'Lung cancer || id:bbj−a−133'
